# Environmental and Genetic Determinants of Major Chronic Disease in Beijing–Tianjin–Hebei Region: Protocol for a Community-Based Cohort Study

**DOI:** 10.3389/fpubh.2021.659701

**Published:** 2021-06-04

**Authors:** Kuo Liu, Han Cao, Chunyue Guo, Li Pan, Ze Cui, Jixin Sun, Wei Zhao, Xiaoyan Han, Han Zhang, Zhengfang Wang, Kaijun Niu, Naijun Tang, Guangliang Shan, Ling Zhang

**Affiliations:** ^1^Department of Epidemiology and Health Statistics, School of Public Health, Capital Medical University, Beijing, China; ^2^Beijing Municipal Key Laboratory of Clinical Epidemiology, Beijing, China; ^3^Department of Epidemiology and Statistics, Institute of Basic Medical Sciences, Chinese Academy of Medical Sciences, Beijing, China; ^4^School of Basic Medicine, Peking Union Medical College, Beijing, China; ^5^Department of Chronic and Noncommunicable Disease Prevention and Control, Hebei Provincial Center for Disease Prevention and Control, Shijiazhuang, China; ^6^Department of Chronic and Noncommunicable Disease Prevention and Control, Chaoyang District Center for Disease Prevention and Control, Beijing, China; ^7^Health Management Center, Beijing Aerospace General Hospital, Beijing, China; ^8^Nutritional Epidemiology Institute and School of Public Health, Tianjin Medical University, Tianjin, China; ^9^Department of Occupational and Environmental Health, School of Public Health, Tianjin Medical University, Tianjin, China

**Keywords:** environmental determinants, public health, genetics, non-communicable disease, risk prediction

## Abstract

**Introduction:** Personal lifestyle and air pollution are potential risk factors for major non-communicable diseases (NCDs). However, these risk factors have experienced dramatic changes in the Beijing–Tianjin–Hebei (BTH) region in recent years, and few cohorts have focused on identifying risk factors for major NCDs in this specific region. The current study is a large, prospective, long-term, population-based cohort study that investigated environmental and genetic determinants of NCDs in BTH areas. The results of this study may provide scientific support for efforts to develop health recommendations for personalized prevention.

**Methods:** About 36,000 participants 18 years or older would be obtained by multistage, stratified cluster sampling from five cities for the baseline assessment. Participants underwent seven examinations primarily targeting respiratory and circulatory system function and filled out questionnaires regarding lifestyle behavior, pollutant exposure, medical and family history, medication history, and psychological factors. Biochemistry indicators and inflammation markers were tested, and a biobank was established. Participants will be followed up every 2 years. Genetic determinants of NCDs will be demonstrated by using multiomics, and risk prediction models will be constructed using machine learning methods based on a multitude of environmental exposure, examination data, biomarkers, and psychosocial and behavioral assessments. Significant spatial and temporal differentiation is well-suited to demonstrating the health determinants of NCDs in the BTH region, which may facilitate public health strategies with respect to disease prevention and survivorship-related aspects.

## Introduction

It is well-known that large cohort studies play essential roles in gaining knowledge about diseases and reducing their associated burden (1). Stroke, ischemic heart disease, and traffic injury are the top three leading causes of years of life lost (YLLs) in China (2). The risk factors contributing to the loss of disability-adjusted life-years include inappropriate dietary habits such as high sodium consumption (15.9%), hypertension (13.3%), tobacco use (12.5%), and air pollution (9.3%) (3). The area around the capital of China, called the Beijing–Tianjin–Hebei (BTH) region, is the political, economic, and cultural center of China. In the BTH area, ischemic heart disease is the leading cause of YLLs, and inappropriate dietary habits result in high sodium consumption. In 2016, the “Healthy China 2030 Plan” was launched by the State Council. According to this plan, health-related risk factors for non-communicable diseases (NCDs) will be under effective control by 2030, including an increase in the proportion of individuals taking physical exercise and a reduction in the per-capita daily salt intake by 20% (4). As residents in the BTH area may experience a significant change in lifestyle risk factors in the next few decades, a cohort study is necessary to explore the effect of health determinant changes on NCDs and to demonstrate the effect of changes due to health-related strategies.

Air pollution is an important trigger of myocardial infarction (MI), and the population attributable fraction of fine particle matter is ~5% (5). Recent systematic reviews and meta-analyses have demonstrated the harmful effect of short-term ambient particle matter and gaseous pollutants [NO_2_, carbon monoxide (CO), CO_2_] on MI (6), stroke (7), and arrhythmia (8), but the evidence is less consistent for ozone (O_3_). Several studies in Western countries have evaluated the adverse health effects of long-term PM_2.5_ exposure and have demonstrated that long-term exposure to even low PM_2.5_ concentrations (PM_2.5_ ranging from 6.3 to 13 μg/m^3^) could promote cardiovascular events and increase cardiovascular mortality (9–11). In China, long-term exposure to air pollution has been shown to be associated with a higher prevalence of cardiometabolic risk factors, especially hyperbetalipoproteinemia (12). Another study from China demonstrated that long-term exposure to PM_2.5_ was independently associated with incident hypertension at relatively high ambient concentrations (13). Although various studies have demonstrated the adverse health effects of ambient air pollutants, some of them did not take potential indoor air pollution into consideration, which may lead to overestimation of the effect size. A study in Beijing showed a dose–response relationship between short-term PM_2.5_ exposure (PM_2.5_ ranged from 3.9 to 494 μg/m^3^) and ischemic heart disease (IHD) morbidity and mortality that was supralinear (14). However, evidence on the dose–response relationship between long-term exposure to air pollution and health effects is still limited. The full dose–response curve needs to be better elucidated because of its huge impact on global public health. In the current cohort study, regions with different concentrations of air pollution exposure were selected as survey sites, which can provide evidence about the dose–response relationship between long-term pollution exposure and NCDs.

Fine particulate matter (PM_2.5_) exposure contributes to high mortality in Beijing (15). The overall concentration of pollutants from northwest to southeast is gradually increased in the BTH region. In the BTH region, some cities, such as Baoding and Shijiazhuang, have extremely high concentrations of PM_2.5_ (16), whereas some cities, such as Chengde, have good natural environments and air quality. This geographical distribution of pollutants will generate natural exposure groups, which can be used to explore the role of pollutants in NCD pathology. To address heavy pollution in China, the Air Pollution Prevention and Control Action Plan was issued in 2013 by the State Council of China. With the implementation of the Action Plan, the emissions of sulfur dioxide (SO_2_), nitrogen oxide (NO_X_), and PM_2.5_ decreased by >30% in 2017 compared with the emissions in 2012 in the BTH region (17, 18). Thus, the severity of air pollution varies not only among regions but also over time. Because of the significant spatial and temporal differentiation of pollutant distribution, studying a cohort in the BTH region will play an essential role in demonstrating the effect of pollutants on the etiology of chronic disease.

Cohorts based on natural communities will improve the understanding of a wide range of risk factors for NCDs. The prospective China Kadoorie Biobank (CKB) is the largest cohort in China. Previous studies have demonstrated many lifestyle, environmental, and genetic risk factors for chronic diseases in the Chinese population (19–22). However, the determinants of disease consist of genetic, epigenetic, environmental, and lifestyle risk factors that interact with one another and operate within the larger physical-sociocultural environment. Thus, it is unrealistic to find a common effective solution for disease prevention. The BTH region is not included in the CKB cohort. However, as the BTH region has a unique socioeconomic status (SES) and political function, it is important to find health determinants of NCDs in this specific region.

In recent years, many policies have been introduced and implemented in the BTH region that affect lifestyle, SES, and air pollution. In 2017, the Xiong'an New Area was established in Hebei Province to evacuate some industries from Beijing, such as the high-energy consumption industries and enterprises with less innovation and technology. The Xiong'an New Area mainly includes three counties in Hebei Province, which previously were mostly agricultural areas (23). The municipal government of Beijing, some tertiary hospitals, universities, large wholesale markets, and factories are moving from the urban area of Beijing to its suburbs to reduce traffic congestion and the severity of air pollution. These strategic changes in the BTH region will have a tremendous impact on the SES of residents and will gradually improve the air quality. Therefore, establishing a community-based cohort in the BTH region will be especially effective in exploring the impact of SES and air pollution on common chronic diseases.

## Methods and Analysis

### Objectives

- To monitor the epidemic trends of major NCDs.
- To demonstrate the etiology and pathogenesis of major chronic diseases with consideration of environmental, lifestyle behavior, genetic, and psychological factors.
- To screen biomarkers of air pollution–related cardiopulmonary diseases and construct disease risk prediction models.

### Outcomes of Interest

- Number of deaths and cause of death.
- Incident of major NCDs, such as hypertension (*ICD-10*: I10-I15), diabetes (*ICD-10*: E10-E14), ischemic heart disease (*ICD-10*: I20-I25), cerebrovascular disease (*ICD-10*: I60-I69), chronic obstructive pulmonary disease (*ICD-10*: J44), and cancer (*ICD-10*: C00-C97).
- Intermediate phenotypes or key risk factors for NCDs, including anthropometric factors and physiological, lifestyle, and environmental factors.

### Study Design and Settings

The CoHort study on CHronic disease of Community Natural population in Beijing–Tianjin–Hebei region (CHCN-BTH) is a population-based prospective cohort study, which has been registered with the Chinese Clinical Trial Registry (ChiCTR1900024725).

**Figure 1:**
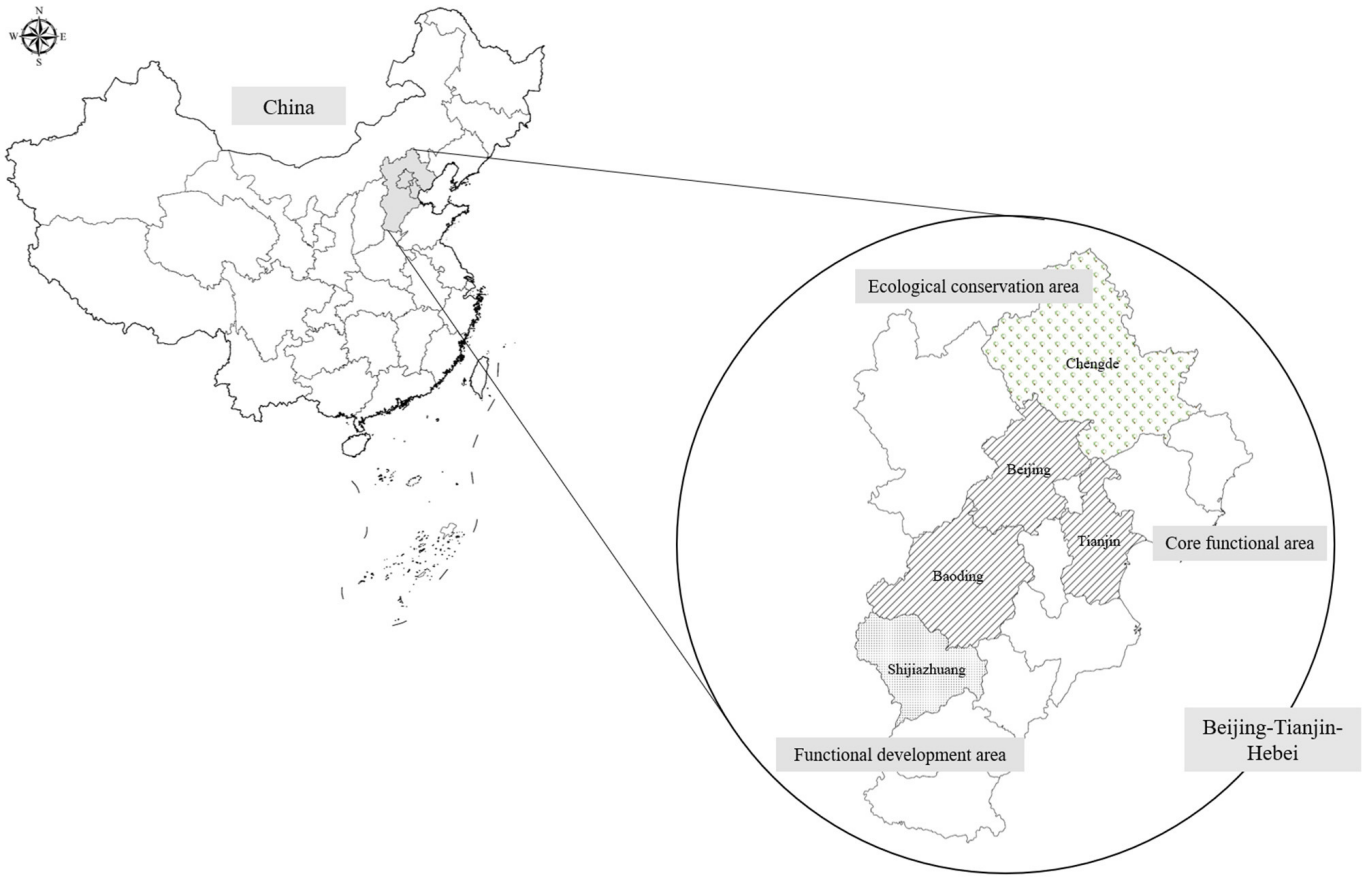
Sampling set of CHCN-BTH.

The sample size was estimated using the Cox regression module in PASS-NCSS 11.0 software. The parameters are as follows: statistical power is 80%, significance level is 0.05, and the lowest event rate is 50 per 100,000 (cardiovascular morbidity is ~650 per 100,000, and mortality is ~250 per 100,000; lung cancer morbidity is ~70 per 100,000, and mortality is 50 per 100,000). The log hazard ratio (HR) of PM_2.5_ per 10 μg/m^3^ is ~0.25 (HR = 1.28), the standard deviation of PM_2.5_ (per 10 μg/m^3^) is 1.4, and the *R*^2^ of PM_2.5_ with other covariates was set to 0.2. The lowest sample size was 32,037 according to the calculation of PASS software. Thus, the cohort was designed to recruit 36,000 individuals living in Beijing, Tianjin, and Hebei provinces by expecting a 10% loss to follow-up. The survey sites are shown in Figure 1. The CTCH-BTH cohort will include 30,000 community-lived individuals and 6,000 participants who undergo routine physical examinations. Most of the participants in the baseline survey were of the Chinese Han population, and ~10% of the participants were of Manchu ethnicity.

### Sampling Methods

**Figure 2:**
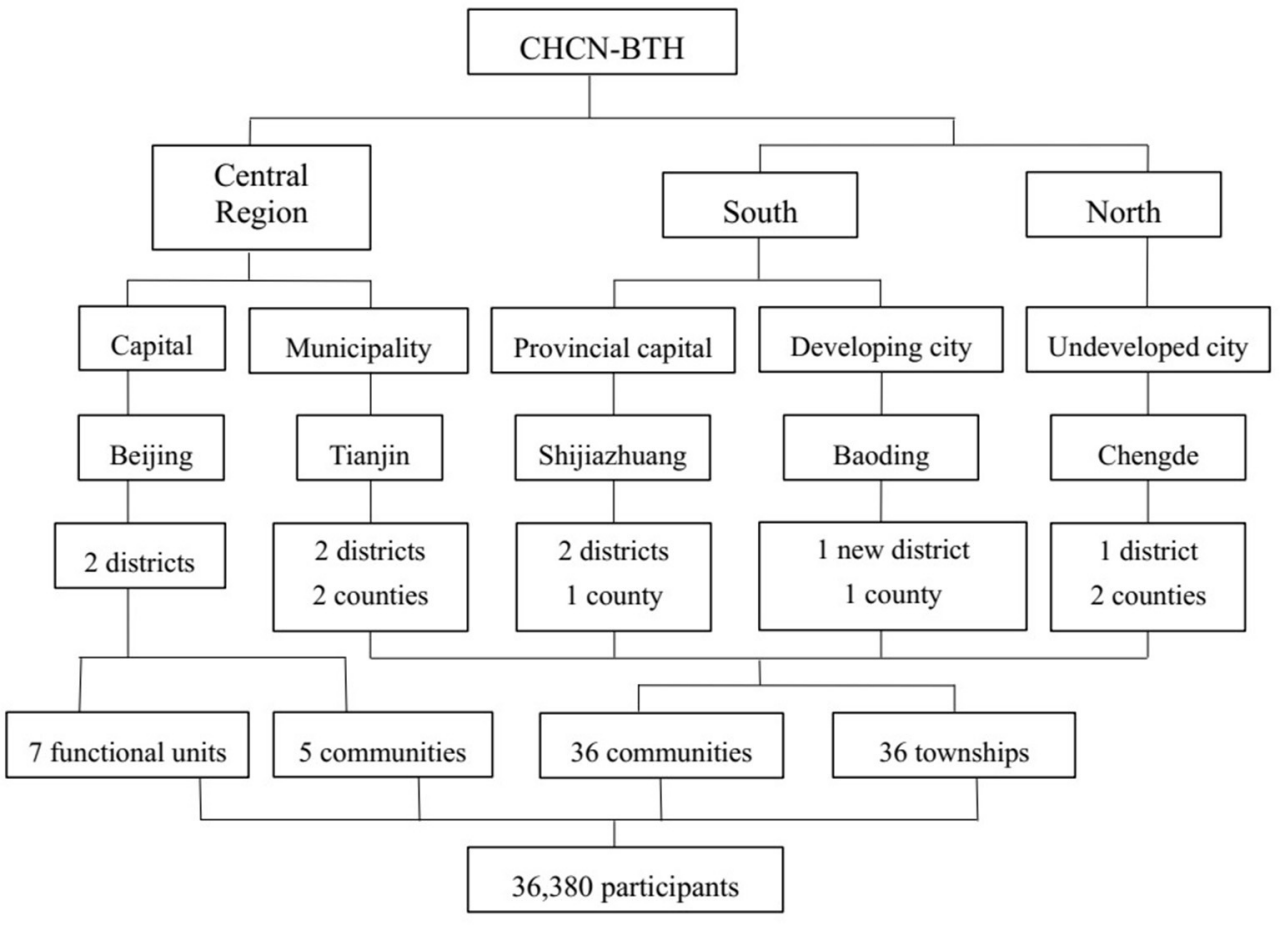
Sampling flowchart of CHCN-BTH.

The cohort study was established with a multistage, stratified cluster sampling method. The cities are first selected according to geographical region, SES, and medical service quality. In the first stage, three cities were chosen as survey sites located in the southern, central, and northern parts of Hebei Province. Beijing and Tianjin are also designated survey sites. Among the above survey sites, the severity of air pollution and economic status are significantly different from each other, which can reflect the state of the natural population in Hebei Province. In the second stage, we selected districts from urban cities and counties from rural areas. In the next stage, communities were selected from districts in urban areas, while townships were selected from counties in rural areas. The new district called Xiong'an, which previously contained three rural counties, has now integrated into one urban city. In the final stage, residents in the selected area will be invited to participate in the study considering the demographic structure of the sixth national census. These procedures can ensure the representation of the sampling. Finally, the selected survey sites included 41 communities, 36 townships, and 7 functional units in five cities. Details of the sampling are shown in Figure 2.

### Ethical Consideration

This study was approved by the ethics committee of the Center of Disease Control (IRB2017-003, CYCDPCIRB-20170830-1) and Capital Medical University (2018SY81). Written informed consent was obtained from all participants prior to the baseline survey.

### Participants and Selection Criteria

To establish a community-based cohort study in the BTH area, participants will be eligible for the study if they meet the following inclusion criteria: (1) ≥18 years old; (2) living in the local area for more than 3 years; (3) can complete the investigation independently or with the help of investigators; and (4) signed informed consent. Participants were excluded from the study if they met any of the following exclusion criteria: (1) pregnant women; (2) patients with major physical or mental disabilities that might invalidate informed consent or the interview outcomes according to clinical judgment; and (3) unable to complete the baseline survey even with the help of investigators. All of the participants meeting the above criteria will undergo a physical examination after fasting for 8 h and be recruited for the CHCN-BTH study.

### Measurements

**Figure 3:**
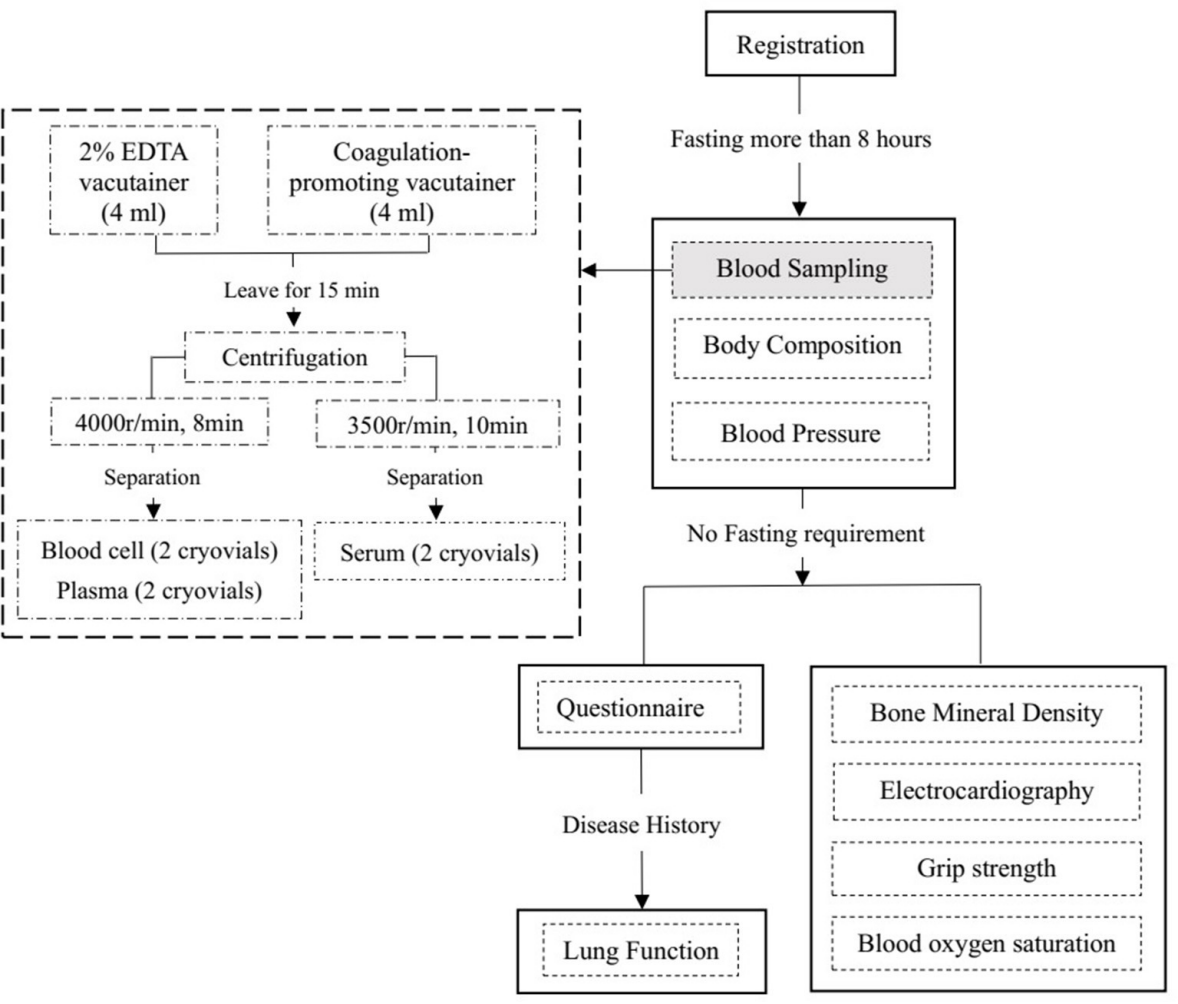
Flowchart of the baseline survey in CHCN-BTH.

Participants were required to undergo fasting blood sampling, blood pressure (BP) and body composition measurements first. All of these tests were conducted after fasting for at least 8 h. Then, the participants will undergo measurements of bone mineral density and electrocardiography (ECG) and have a face-to-face interview to complete the questionnaire. In addition to the information about the disease history in the questionnaire, lung function measurements were performed in participants except for those meeting the following exclusion criteria: (1) underwent chest, abdomen, or eye surgery in the last 3 months; (2) had a heart attack (angina pectoris, MI, or severe arrhythmia) in the last 3 months; (3) systolic BP (SBP) ≥140 mmHg or diastolic BP (DBP) ≥90 mmHg on the day of the survey; (4) history of tuberculosis, disinsertion, tumor, schizophrenia, or cognitive disorder; or (5) pregnant or lactating women. Table 1 provides an overview of all study assessments. A flowchart of the field survey is shown in Figure 3.

**Table 1:** Study timeline and assessment of the CHCN-BTH.

	**Baseline**	**Follow-up^£^**	Repeated measurements^*^
**Baseline characteristics**			
Sociodemographic data	X	X	X
Personal and family disease history	X		
Lifestyle behaviors	X	X	X
Indoor air pollution exposure	X		X
Mental health	X		X
Blood pressure	X	X	X
Body composition	X		X
Electrocardiography	X		X
Bone mineral density	X		X
Lung function	X		X
Grip strength	X		X
Oxygen saturation	X		X
Serum markers	X	X	X
**Outcome measures**			
Hypertension		X	X
Diabetes		X	X
Ischemic heart disease		X	X
Cerebrovascular disease		X	X
Chronic obstructive pulmonary disease		X	X
Cancer		X	X
Death/cause of death		X	X

£ *All the participants will be followed up every 2 years; the proportion of loss to follow-up will be restricted to <10%*.

* *A subgroup of baseline participants will be selected to do repeated measurement every 2 years; the minimum proportion of repeated measurements will be 20% in each time of follow-up. All measures will be repeated after five patient follow-ups*.

Suitable places for the field survey were selected after consideration of the space needed for the survey instruments and the waiting space needed for the survey subjects. For example, we selected community healthcare centers or temporarily requisitioned schools near community healthcare centers as survey places. Face-to-face interviews will be performed using a uniform questionnaire. To ensure the accuracy of the questionnaire information, all questionnaires will be verified by the inspectors. The questionnaire includes five sections: sociodemographic characteristics, familial and personal medical history, lifestyle behaviors (including drinking, smoking, physical exercise), pollutant exposure (passive smoking condition and air pollution exposure condition), and psychological conditions. Current smoking was defined as smoking at least one cigarette per day in the last 6 months. Former smoking was defined as smoking less than one cigarette per day for at least 6 months. Alcohol drinking was defined as accumulatively drinking wine/liqueur more than 250 g/year or drinking beer more than 5 L/year (24). Physical exercise was defined as participating in moderate or vigorous activity for at least 30 min each time and at least once per week. Information on psychological conditions was collected using the Depression, Anxiety and Stress Scale-21 (25, 26). The scale includes three subscales, and each subscale contains seven items. The details of the questionnaire are shown in Table 2.

**Table 2:** Sections and topics of the questionnaire.

**Type of data**	**Components**
Sociodemographic characteristics	Sex, age, ethnicity
	Residence address
	Cellphone number
	Personal income, education, occupation
	Menstrual history
Personal and family disease history	Hypertension (date of diagnosis, medication)
	Diabetes (date of diagnosis, medication)
	Cardiovascular disease (date of diagnosis, medication)
	Respiratory disease
	Gastrointestinal disease
	Cancer
	Others
Lifestyle behaviors	Tobacco use and smoking
	Alcohol consumption
	Labor intensity
	Physical activity/frequency
Outdoor exposure	Outdoor activity/frequency
	Window opening frequency
	Protective measurements during smog:
	Stay indoors/mask wearing/usage of air purifier
Indoor air pollution exposure	Passive smoking
	Cooking fuel type, cooking frequency
	Cooking habits:
	Kitchen door opening/usage of smoke extractors
	Winter heating fuel type
Mental health	Depression (normal: ≤ 9; mild: 10–13; moderate: 14–20; severe: 21–27; serious severe: ≥28)
	Anxiety (normal: ≤ 7; mild: 8–9; moderate: 10–14; severe: 15–19; serious severe: ≥20)
	Stress (normal: ≤ 14; mild: 15–18; moderate: 19–25; severe: 26–33; serious severe: ≥34)

Subjects will be asked to bring the packaging of their most recent medications, as well as any medical records from secondary or tertiary hospitals, before they participate in the baseline survey. When conducting the face-to-face questionnaires, investigators with medical backgrounds will check the participants' medical records and their medicine packages, and then they will record the participants' disease history and medication history according to their findings. For self-reported incident diseases during the follow-up, the staff of the community health service center will collect the patient's medical information, including hospital, hospital admission time, the results of medical examinations, and diagnoses. The Clinical Event Committee (CEC) comprised three to five physicians working in tertiary hospitals with relevant specialties (hypertension, diabetes, cardiovascular disease, chronic obstructive pulmonary disease, and cancer). CEC members will ascertain the outcome events according to the medical records provided by the participants or collected by community healthcare workers.

Indoor pollutant exposures will also be obtained from face-to-face interviews, including asking about passive smoking, cooking fuel, cooking habits, and winter heating fuel. Ambient air pollutants are generally estimated by the following procedure: (1) annual concentrations of particles with aerodynamic diameters of no >2.5 μm (PM_2.5_) will be estimated with a machine learning method at a resolution of 0.1° × 0.1° using high-dimensional expansion of numerous predictors (including ground-monitored PM_2.5_ data, satellite-derived aerosol optical depth and other satellite covariates, meteorological variables, and chemical transport model simulations). (2) Concentrations of particles with aerodynamic diameters of no >10 μm (PM_10_), nitrogen dioxide (NO_2_), SO_2_, CO, and O_3_ will be collected using air monitoring stations. In each of the study districts, there will be more than one air monitoring station. Continuous hourly concentrations will be gathered. The daily average concentrations of PM_2.5_, PM_10_, SO_2_, NO_2_, and CO at each station will be used only if >20 of the 24-hourly measurements are available. For O_3_, at least six hourly concentrations of O_3_ per day will be needed to calculate the 8-h average concentration of O_3_. The air pollutant concentration measurements are valid and are conducted in accordance with the China Ambient Air Quality Standards (GB 3095-2012). Information on outdoor activity, window opening frequency, and protective measurements during smog are also included in the questionnaire to evaluate the effect of ambient air pollutants more accurately.

Physical examinations will include assessments of BP, heart rate, grip strength, oxygen saturation, body composition, bone mineral density, ECG, and lung function. BP will be measured with a digital sphygmomanometer (OMRON HEM-907, Japan) after resting in a seated position for at least 10 min. Measurements will be recorded three times with 1-min intervals, and the mean SBP and DBP will be recorded. Body composition will be measured in the upright position after fasting and urinating with their arms naturally hanging by using a body composition analyzer (TANITA BC-420, Japan). Resting ECG will be performed in the supine position after sitting quietly for 5 min. A standard 12-lead ECG will be acquired digitally using a Mortara ECG machine (Mortara Eli 250c, Milwaukee, WI, USA) at a calibration of 10 mm/mV and a speed of 25 mm/s. Bone mineral density will be measured in a seated position using an Osteospace Pegasus ultrasound bone densitometer (MEDILINK, France). Bone mineral density measurements will be conducted on the participant's bare right ankle and foot by using a Pegasus device (MEDILINK, France). Lung function measurement (spirometry) will be conducted using a portable spirometer (CareFusion MasterScreen Pneumo, Germany) according to the guidelines issued by the American Thoracic Society (27). All physical examinations will be carried out by trained staff based on a standard manual, and all of the measurement equipment will be regularly calibrated. Details of the physical examination measurements are shown in Table 3.

**Table 3:** Physical examination measurements and laboratory test.

**Measures**	**Measuring equipment**	**Variables**	**Times of measurements**
Blood pressure	OMRON HEM-907, Japan	Systolic pressure (SBP, mmHg), diastolic blood pressure (DBP, mmHg)	3
Body composition	TANITA BC-420, Japan	Height, cloth weight, weight, reference value of weight, fat mass rating (fat%), reference value of fat%, fat mass, reference value of fat mass, fat-free body mass, muscle mass, reference value of muscle mass, muscle mass rating, bone mass, reference value of bone mass, body water, reference value of the body water, the body water rating, reference value of the body degree, body mass index (BMI), standard weight, obesity degree, visceral fat rating, the basic metabolic rate, the basic metabolic rate, basal metabolism, metabolism age, and impedance	1
Electrocardiography	Mortara Eli 250c, Milwaukee, WI, USA	Heart rate, basic heart rhythm, PR interval, QRS complex, QT interval, QTc interval, P axis, and T axis	1
Bone mineral density	MEDILINK, France	Bone mineral density	
Lung function	CareFusion MasterScreen Pneumo Germany	Forced vital capacity (FVC), forced expiratory volume in 1 s (FEV1), peak expiratory flow (PEF), maximum midexpiratory flow (MMEF), and FEV1:FVC ratio	
Grip strength	JAMAR Kit, UK	Grip strength (kg)	2
Oxygen saturation	Masimo Red-57, USA	Oxygen saturation (%)	2
Serum markers	Beckman coulter chemistry analyzer AU5800	Aspartate aminotransferase (AST, U/L), alanine aminotransferase (ALT, U/L), γ-glutamyl transferase (GGT, U/L), urea (mmol/L), creatinine (CrE, μmol/L), uric acid (UA, μmol/L), fasting plasma glucose (FBG, mmol/L), cholesterol (TC, mmol/L), triglycerides (TG, mmol/L), high-density lipoprotein cholesterol (HDL-C, mmol/L), low-density lipoprotein cholesterol (LDL-C, mmol/L), hsCRP (mg/L), and Lp(a) (mg/L)	1

Fasting blood samples will be collected into a 2% EDTA Vacutainer and a coagulation-promoting Vacutainer. After centrifugation and aliquoting, four cryovials (including two plasma and two blood cell samples) will be filled from the 2% EDTA Vacutainer, and the serum samples from the coagulation-promoting Vacutainer will be separated into two cryovials. All of the above samples will be kept in an insulated box with ice packs to maintain their temperature at 0–4°C and transported to the local Center of Disease Control within 4 h. After being stored at −20°C in the local CDC for a few days, the serum samples will be transported on dry ice to Beijing Hepingli Hospital to test for serum markers. Serum markers will be tested using a Beckman Coulter chemistry analyzer AU5800 in the clinical laboratory of Beijing Hepingli Hospital. By the end of the serum testing, 36,000 sample aliquots will be stored in two geographically separate −80°C archives: 6,500 sample in Chinese Academy of Medical Sciences, and 29,500 sample in Capital Medical University. In each storage site, four cryovials (including two plasma and two blood cell samples) for each participant will be stored in two separate −80°C fridges (28).

### Follow-Up

Community-based participants will be followed up every 2 years. All of the participants will be followed up by telephone-based interviews. Several methods will be used to achieve the goal that the overall follow-up rate should be more than 90%. Ways to guarantee a high follow-up rate are as follows: (1) For each participant, we will conduct telephone interviews on both workdays and weekends to ensure they can answer the telephone at least once. (2) For participants who cannot be contacted by telephone, a household survey will be conducted according to their baseline home address. (3) For participants who could not be contacted by telephone or household surveys, medical records in community healthcare centers will be acquired to confirm their health status and to collect information on BP, weight, and fasting blood glucose. (4) Death surveillance is generally based on the Disease Surveillance Point (DSP) system. For places not covered by DSPs, community healthcare centers will help to collect medical certificates of death. Photos of death certificates will be named “ID+name of participants” and collected by the research group. (5) Subjects will be deemed lost to follow-up if they cannot be contacted by telephone or household survey, and their medical or death records are not accessible. Information on home address changes, self-reported disease status, and its potential determinants will be collected in the follow-up survey through telephone interviews or household surveys. The expected proportion of participants lost to follow-up is <10%. We will recruit new participants in their community to replace the lost participants, matching by age and sex.

**Figure 4:**
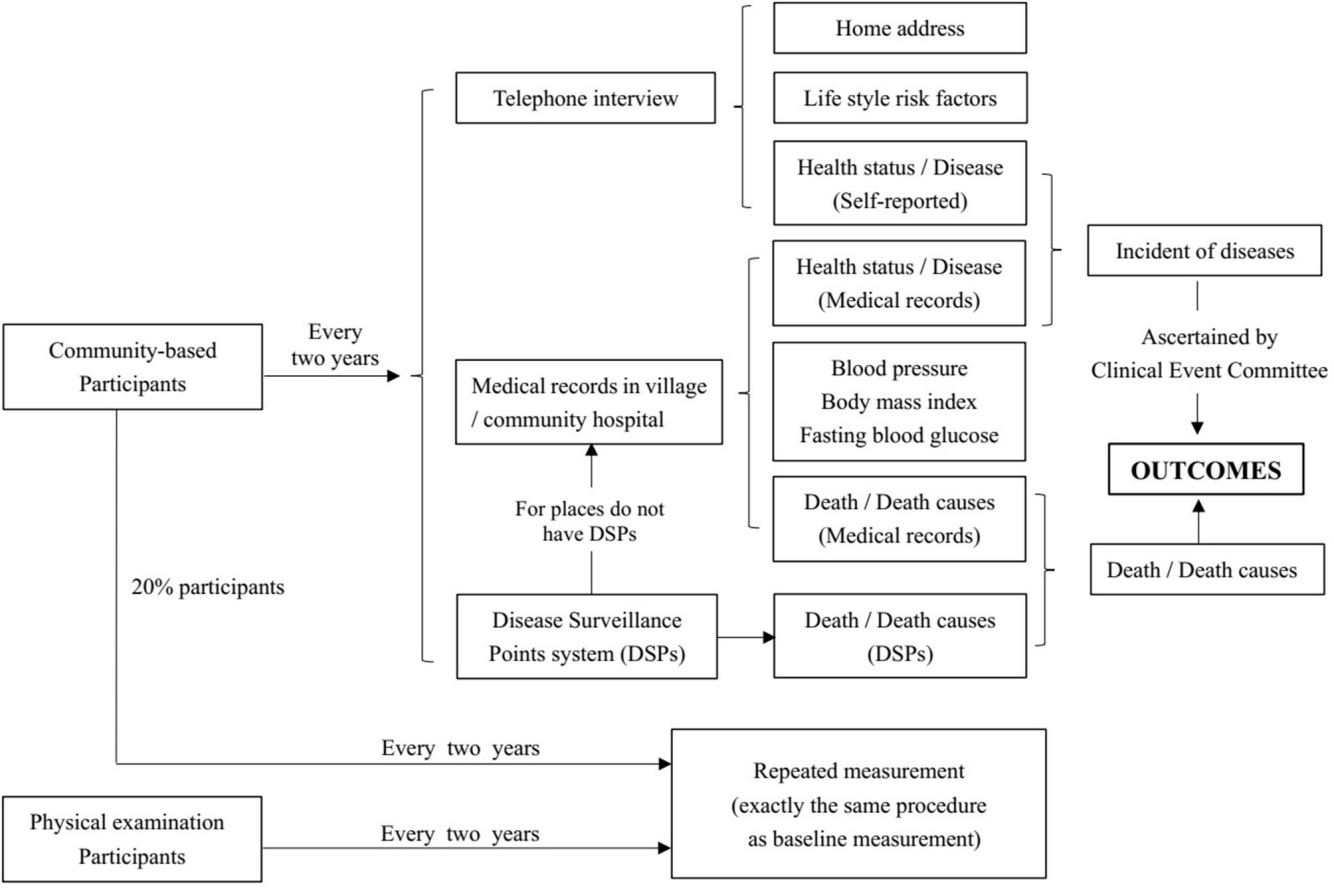
Follow-up flow diagram in CHCN-BTH.

Among the participants interviewed by telephone in the follow-up survey, we will randomly select 1% to conduct the survey again at each survey site. If the proportion of inconsistent information from the above two surveys is >10%, all of the participants at that survey site will be resurveyed. For all the community-based participants, BP, disease status, and serum markers (fasting glucose, total cholesterol, triglyceride, low-density lipoprotein cholesterol, and high-density lipoprotein cholesterol) will be accessed through medical records in community hospitals during follow-up survey. The minimum proportion of repeated measurements among community participants will be 20% at each follow-up. All participants will be examined after five rounds of follow-up surveys. For participants who undergo employee physical examinations, serum markers and physical examination exactly the same as baseline will be examined every 2 years. The follow-up procedure is shown in Figure 4.

### Statistical Plan

All potential risk factors and outcomes will be described at baseline and at each follow-up. Normally distributed continuous variables will be summarized by the mean and standard deviation, and skewed distributed continuous variables will be described by the median and interquartile range. To compare possible differences between two groups, an independent-samples *t*-test will be used for qualitative variables with a normal or skewed distribution, and the χ^2^-test will be used for categorical variables. The χ^2^-test will be used for categorical variables in the description of the profiles of the responders and non-responders. When comparing differences among more than two groups, the Kruskal–Wallis test will be used for continuous variables, and one-way analysis of variance will be used for quantitative variables. Trajectory analysis will be used to explore the effect of risk factor changes on NCDs.

Generalized additive mixed models will be used to examine the associations of long-term air pollutant exposures with physiological indicators and the prevalence of NCDs. The exposure assessment during the follow-up period will use a time-spatial continuous prediction model. Time-dependent Cox proportional hazards models will be used to analyze the associations of air pollutant exposures with the incidence of NCDs. A penalized cubic regression spline of air pollutants will be included in the models to explore the non-linear exposure–response relationships.

To screen for biomarkers and genetic markers of NCDs, lasso regressions will be used first, and then mixed models will be used to demonstrate their effect size in the longitudinal data. Logistic regressions and generalized linear models will also be used to confirm the effect of potential risk factors on NCDs. To demonstrate a genetic susceptibility to NCDs, single-nucleotide polymorphism (SNP), lncRNA, and mRNA expression profiles will be detected using a microarray. SNPs associated with diseases will be explored by logistic regression, and haplotypes will be identified by using Plink software. Gene–gene and gene–environment interactions will be demonstrated by using generalized multifactor dimensionality reduction and the random forest (RF) method. Differentially expressed genes (DEGs) will be identified using the R software Limma package (29). The lncRNA–mRNA coexpression network will be constructed based on the expression level of DEGs using weighted gene coexpression network analysis (30, 31). MiRNAs targeted by both lncRNAs and mRNAs in the lncRNA–mRNA coexpression network will be predicted by miRDB, TarBase, and TargetScan. All of the ceRNA loops will be combined and transformed into an “lncRNA–miRNA–mRNA” ceRNA network using Cytoscape software (32).

To construct risk prediction models, lasso regression, smoothly clipped absolute deviation, RF, support vector machine, and decision tree will first be used to screen for predictors of NCDs. Then, predictors selected by machine learning methods will be used to construct different prediction models by the Cox regression model. The Cox regression model with the best discrimination and calibration will be selected as the risk prediction model.

## Discussion

The current study emphasizes the need for cohort studies in China, especially in the BTH region, to understand NCDs and their risk factors. China is a very diverse country with different ethnic minorities, SESs, and rates of urbanization; thus, the risk factors for non-communicable conditions can vary widely from one geographical area to another. The prospective CKB, which includes more than 0.5 million adults, is the largest cohort in China. However, this cohort did not include a population in the BTH region. To our knowledge, the current study is the first large cohort study based on a community population in the BTH region. The current cohort is conducted in a centrally coordinated manner, which is similar to several cohort studies, such as LifeGene (33), National Health and Nutrition Examination Survey (34), and UK biobank (28). Central coordination center is located in Beijing and is composed of researchers from Capital Medical University and the Chinese Academy of Medical Sciences. The central coordination center is responsible for the quality control, training certification, biological sample storage, providing unified medical examination equipment, test center selection, participant recruitment, organizing CEC to ascertain outcomes, and proposing follow-up plan. Local CDC will assist in baseline field investigation and follow-up investigation.

China is a multiethnic country. There are 55 minorities in China in addition to the Chinese Han population. Most of the cohorts in China only contain the Han population. We will recruit not only a Chinese Han population in the cohort but also a Manchu population of ~10%. Manchu is one of the most populous minorities in the BTH region. Based on the sixth China national population census, Manchu was the largest minority group in Hebei (3.02%) (35). The Manchu population prefers sticky foods and a high-salt diet, and their prevalence of several diseases, such as hypertension and diabetes, is different from the Han population (36). Studies focusing on both Manchu and Han populations may help us to find more specific determinants of chronic disease in the BTH region. Moreover, the genetic background of the Manchu population is also different from that of the Han population (37). Given that population stratification will lead to spurious results and poor reproducibility in genetic association findings, grouping individuals into ethnic clusters will be effective in controlling population heterogeneity.

Classic cohort studies generally focus on certain types of exposure factors and certain types of diseases. The current cohort will not only focus on traditional lifestyle risk factors but also investigate dietary habits, mental health, environment, genetics, and epigenetics so that the etiology of common chronic diseases can be demonstrated comprehensively. Given that many chronic diseases share common pathogenic pathways, the current cohort will systematically analyze multiple exposures and their effects on diseases based on genomics, proteomics, and metabolomics to identify the specific and shared pathways of related diseases. For data collection, in addition to those based on previous traditional chronic disease management modes, this project will explore new methods to achieve real-time dynamic data monitoring and automatic data collection. For example, we will use an ID card reader to collect the subject's ID number, sex, birthday, and living address automatically and save the above information in an Excel table. The participants will be identified by the patient's ID number from the health management records in community healthcare centers, and their medical information will be collected and updated dynamically. Electronic information acquisition systems based on mobile terminals will play important roles not only in obtaining health-related big data but also in health management in the community. Moreover, 20% of the participants will be repeatedly measured in the follow-up visits, which offers an opportunity to conduct trajectory analyses to determine the dynamic patterns of risk factors contributing to the disease.

There are several limitations of the current cohort. First, the survey sites and the participants will not be randomly chosen. This is because the current study focuses on selected survey sites with various economic, medical, and environmental characteristics to better demonstrate the pathogenic mechanism of NCDs. In the central functional district of Beijing, we selected Chaoyang district. Tianjin and Shijiazhuang are in good terms of social economics with moderate air pollution, while Baoding is a city with moderate economic status and severe air pollution. Chengde city was selected as having better air quality. Xiong'an city may experience rapid economic development in the next 5 years. The residents' socioeconomic level and lifestyle may undergo tremendous changes, which may have a certain impact on health. Moreover, instead of estimating the prevalence of diseases by representative sampling, this cohort study is more concerned about the proportion of participants who could be followed up. Thus, we chose stable communities and participants to ensure a good follow-up rate. During the study recruitment process, the investigator will explain that enrollment in the cohort requires continued follow-up. Subjects who actively choose to join the cohort may have a stronger willingness to participate compared with randomly chosen participants, which may avoid loss to follow-up. Second, the current cohort will exclude participants with major physical and mental disabilities and any who could not complete the survey even with the help of investigators. These exclusion criteria may lead to a “healthy cohort” effect, and the results of this cohort study will be more applicable to a healthier population and should be explained and extrapolated with caution. Third, most of the surveys will be conducted on workdays, and elderly people and retirees may prefer to participate compared with young people and employees, which may introduce selection bias. To reduce selection bias and better represent the target population, we will recruit participants considering their demographic structure according to the results of the sixth national census. Finally, the same examination equipment will be used for physical examination to ensure the comparability of measurements. Because of the limited quantity of equipment, it is not possible to carry out repeated measurements at the same time. Therefore, in this study, only 20% of the subjects were repeated measured in each time of follow-up. It was planned to complete repeated measurements of all the subjects after five rounds of follow-up. In order to overcome the shortage of partial repeated measurements, telephone interviews and medical records update will be conducted to all participants every 2 years to obtain the change of lifestyle behavior and other health determinants.

## Conclusion

The CHCN-BTH cohort will be a combination of self-reported data, medical records, and biological information. These data will contribute to our knowledge of risk factors for major NCDs and their survivorship. The survey sites of the CHCN-BTH cohort will have different socioeconomic and environmental statuses, and their socioeconomic and environmental status have experienced dramatic changes in recent decades. Significant spatial and temporal differentiation is well-suited to demonstrate the health determinants of NCDs in the BTH region, which will feed into public health strategies with regard to disease prevention and survivorship-related aspects.

## Ethics Statement

The studies involving human participants were reviewed and approved by Center for Disease Control and Prevention (CYCDPCIRB-20170830-1, Beijing, China; IRB2017-003, Hebei, China) Capital Medical University (2018SY81, China). The patients/participants provided their written informed consent to participate in this study.

## Author Contributions

KL will supervise the field survey, will be responsible for general quality control, and drafted this article. HC will participate in recruiting participants and controlling the quality of lung function measurements, bone mineral density measurements, and ECG. CG will be in charge of blood sample separation and preservation, and in ensuring the quality of BP and body composition measurements. LP will be responsible for the quality control of the questionnaire interview and the consistency of the physical examinations. ZC and JS will organize the field survey in Hebei Province and are responsible for the follow-up in Hebei. WZ and XH will be in charge of the organization of the field survey in Chaoyang, Beijing. HZ and ZW will be responsible for the field survey and follow-up in Fengtai, Beijing. KN and NT will organize and conduct the field survey in Fengtai District in Tianjin and will take charge of its follow-up. GS and LZ designed the study, and directed its implementation and quality control. LZ also helped to review and revise the draft of this manuscript. All authors contributed to the article and approved the submitted version.

## Conflict of Interest

The authors declare that the research was conducted in the absence of any commercial or financial relationships that could be construed as a potential conflict of interest.

## References

[1] Gaziano JM. The evolution of population science: advent of the mega cohort. JAMA. (2010) 304:2288–9. 10.1001/jama.2010.1691 21098773

[2] Zhou M Wang H Zeng X Yin P Zhu J Chen W. Mortality, morbidity, and risk factors in China and its provinces, 1990-2017: a systematic analysis for the Global Burden of Disease Study 2017. Lancet. (2019) 394:1145–58. 10.1016/S0140-6736(19)30427-1 31248666 PMC6891889

[3] Zeng X Qi J Yin P Wang LJ Liu YN Liu JW. Disease burden report of provincial administrative regions in China from 1990 to 2016 [in Chinese]. Chin Circ J. (2018) 33:1147–58. 10.3969/j.issn.1000-3614.2018.12.002

[4] Fu W Zhao S Zhang Y Chai P Goss J. Research in health policy making in china: out-of-pocket payments in healthy China 2030. BMJ. (2018) 360:k234. 10.1136/bmj.k234 29437565 PMC5797981

[5] Nawrot TS Perez L Künzli N Munters E Nemery B. Public health importance of triggers of myocardial infarction: a comparative risk assessment. Lancet. (2011) 377:732–40. 10.1016/S0140-6736(10)62296-9 21353301

[6] Mustafic H Jabre P Caussin C Murad MH Escolano S Tafflet M. Main air pollutants and myocardial infarction: a systematic review and meta-analysis. JAMA. (2012) 307:713–21. 10.1001/jama.2012.126 22337682

[7] Shah ASV Lee KK McAllister DA Hunter A Nair H Whiteley W. Short term exposure to air pollution and stroke: systematic review and meta-analysis. BMJ. (2015) 350:h1295. 10.1136/bmj.h1295 25810496 PMC4373601

[8] Song XP Liu Y Hu YL Zhao XY Tian JH Ding GW. Short-term exposure to air pollution and cardiac arrhythmia: a meta-analysis and systematic review. Int J Environ Res Public Health. (2016) 13:642. 10.3390/ijerph13070642 27367707 PMC4962183

[9] Pinault L Tjepkema M Crouse DL Weichenthal S van Donkelaar A Martin RV. Risk estimates of mortality attributed to low concentrations of ambient fine particulate matter in the Canadian community health survey cohort. Environ Health. (2016) 15:18. 10.1186/s12940-016-0111-6 26864652 PMC4750218

[10] Weichenthal S Villeneuve PJ Burnett RT van Donkelaar A Martin RV Jones RR. Long-term exposure to fine particulate matter: association with nonaccidental and cardiovascular mortality in the agricultural health study cohort. Environ Health Perspect. (2014) 122:609–15. 10.1289/ehp.1307277 24633320 PMC4050514

[11] Thurston GD Ahn J Cromar KR Shao Y Reynolds HR Jerrett M. Ambient particulate matter air pollution exposure and mortality in the NIH-AARP diet and health cohort. Environ Health Perspect. (2016) 124:484–90. 10.1289/ehp.1509676 26370657 PMC4829984

[12] Yang BY Guo YM Markevych I Qian ZM Bloom MS Heinrich J. Association of long-term exposure to ambient air pollutants with risk factors for cardiovascular disease in China. JAMA Netw Open. (2019) 2:e190318. 10.1001/jamanetworkopen.2019.0318 30848806 PMC6484675

[13] Huang KY Yang XL Liang FC Liu FC Li JX Xiao QY. Long-term exposure to fine particulate matter and hypertension incidence in China. Hypertension. (2019) 73:1195–201. 10.1161/HYPERTENSIONAHA.119.12666 31067193 PMC6656583

[14] Xie WX Li G Zhao D Xie XQ Wei ZH Wang W. Relationship between fine particulate air pollution and ischaemic heart disease morbidity and mortality. Heart. (2015) 101:257–63. 10.1136/heartjnl-2014-306165 25341536

[15] Liu M Huang Y Ma Z Jin Z Liu XY Wang HK. Spatial and temporal trends in the mortality burden of air pollution in China: 2004-2012. Environ Inter. (2017) 98:75–81. 10.1016/j.envint.2016.10.003 27745948 PMC5479577

[16] Deng Q Yang K Luo Y. Spatiotemporal patterns of PM2.5 in the Beijing–Tianjin–Hebei region during 2013–2016. Geol Ecol Landsc. (2017) 1:95–103. 10.1080/24749508.2017.1332851

[17] Cai S Wang Y Zhao B Wang SX Chang X Hao JM. The impact of the “Air Pollution Prevention and Control Action Plan” on PM 2.5, concentrations in Jing-Jin-Ji region during 2012-2020. Sci Total Environ. (2017) 580:197–209. 10.1016/j.scitotenv.2016.11.188 28011024

[18] Li J Liu H Lv Z Zhao RZ Deng FY Wang CF. Estimation of PM mortality burden in China with new exposure estimation and local concentration-response function. Environ Pollut. (2018) 243:1710–8. 10.1016/j.envpol.2018.09.089 30408858

[19] Chen Z Iona A Parish S Chen YP Guo Y Bragg F. Adiposity and risk of ischaemic and haemorrhagic stroke in 0.5 million Chinese men and women: a prospective cohort study. Lancet Global Health. (2018) 6:e630–40. 10.1016/S2214-109X(18)30216-X 29773119 PMC5960068

[20] Qin C Lv J Guo Y Bian Z Si J Yang L. Associations of egg consumption with cardiovascular disease in a cohort study of 0.5 million Chinese adults. Heart. (2018) 104:1756–63. 10.1136/heartjnl-2017-312651 29785957 PMC6241631

[21] Sun X Zheng B Lv J Guo Y Bian Z Yang L. Sleep behavior and depression: findings from the China Kadoorie Biobank of 0.5 million Chinese adults. J Affect Disord. (2018) 229:120–4. 10.1016/j.jad.2017.12.058 29306691 PMC6675597

[22] Millwood IY Bennett DA Holmes MV Boxall R Guo Y Bian Z. Association of CETP gene variants with risk for vascular and nonvascular diseases among Chinese adults. JAMA Cardiol. (2018) 3:34–43. 10.1001/jamacardio.2017.4177 29141072 PMC5833522

[23] Jiang LG Lv PY Feng ZM Liu Y. Land use patterns of the Xiongan New Area and comparison among potential choices of start zone [in Chinese]. Resour Sci. (2017) 39:991–7. 10.18402/resci.2017.06.01

[24] Yang W Lu J Weng J Jia W Ji L Xiao J. Prevalence of Diabetes Among Men and Women in China. N Engl J Med. (2010) 362:1090–101. 10.1056/NEJMc1004671 20335585

[25] Antony MM Bieling PJ Cox BJ Enns MW Swinson RP. Psychometric properties of the 42-item and 21-item versions of The Depression Anxiety Stress Scales in clinical groups and a community sample. Psychol Assess. (1998) 10:176–81. 10.1037/1040-3590.10.2.176

[26] Lovibond PF Lovibond SH. The structure of negative emotional states: comparison of the Depression Anxiety Stress Scales (DASS) with the Beck Depression and Anxiety Inventories. Behav Res Ther. (1995) 33:335–43. 10.1016/0005-7967(94)00075-U 7726811

[27] American Thoracic Society. Standardization of Spirometry, 1994 Update. Am J Respir Crit Care Med. (1995) 152:1107–36. 10.1164/ajrccm.152.3.7663792 7663792

[28] Elliott P Peakman TC UK Biobank. The UK Biobank sample handling and storage protocol for the collection, processing and archiving of human blood and urine. Int J Epidemiol. (2008) 37:234–44. 10.1093/ije/dym276 18381398

[29] Ritchie ME Phipson B Wu D Hu YF Law CW. Limma powers differential expression analyses for RNA-sequencing and microarray studies. Nucleic Acids Res. (2015) 43:e47. 10.1093/nar/gkv007 25605792 PMC4402510

[30] Langfelder P Horvath S. WGCNA: an R package for weighted correlation network analysis. BMC Bioinform. (2008) 9:559. 10.1186/1471-2105-9-559 19114008 PMC2631488

[31] Zhang B Horvath S. A general framework for weighted gene co-expression network analysis. Stat Appl Genet Mol Biol. (2005) 4:17. 10.2202/1544-6115.1128 16646834

[32] Kohl M Wiese S Warscheid B. Cytoscape: software for visualization and analysis of biological networks. Methods Mol Biol. (2011) 696:291–303. 10.1007/978-1-60761-987-1_18 21063955

[33] Almqvist C Adami H-O Franks PW Groop L Ingelsson E Kere J. LifeGene–a large prospective population-based study of global relevance. Eur J Epidemiol. (2011) 26:67–77. 10.1007/s10654-010-9521-x 21104112 PMC7087900

[34] Ezzati TM Massey JT Waksberg J Chu A Maurer KR. Sample design: third National Health and Nutrition Examination Survey. Vital Health Stat 2. (1992) 113:1–35. 10.1161/HYP.0b013e3181f92ef6 1413563

[35] Population Census Office under the State Council. National Bureau of Statistics of China. Tabulation on the 2010 Population Census of the People's Republic of China (2012).

[36] Pan Z Cui J Shan G Chou YY Pan Li Sun ZX. Prevalence and risk factors for pterygium: a cross-sectional study in Han and Manchu ethnic populations in Hebei, China. BMJ Open. (2019) 9:e025725. 10.1136/bmjopen-2018-025725 30796128 PMC6398733

[37] Xing J Adnan A Rakha A Kadirya K Noor A Xuan JF. Genetic analysis of 12 X-STRs for forensic purposes in Liaoning Manchu population from China. Gene. (2019) 683:153–8. 10.1016/j.gene.2018.10.020 30326331

